# Giant Keratinocyte Carcinomas: Clinical, Histopathological, Molecular and Health-System Factors with Therapeutic Implications—A Structured Narrative Review

**DOI:** 10.3390/cancers18203367

**Published:** 2026-10-18

**Authors:** Andrzej Ciborek, Hanna Krauss, Zuzanna Chęcińska-Maciejewska, Andrzej Kazimierz Jaworek

**Affiliations:** 1 Department of Interprofessional Education, Faculty of Medicine and Health Sciences, Calisia University (Uniwersytet Kaliski im. Prezydenta Stanisława Wojciechowskiego), ul. Kaszubska 13, 62-800 Kalisz, Poland; a.ciborek@uniwersytetkaliski.edu.pl; 2 Department of Basic Sciences, Calisia University (Uniwersytet Kaliski im. Prezydenta Stanisława Wojciechowskiego), ul. Kaszubska 13, 62-800 Kalisz, Poland; 3 Department of Food and Nutrition, Faculty of Medicine and Health Sciences, Calisia University, ul. Kaszubska 13, 62-800 Kalisz, Poland; 4 Department of Dermatology, Jagiellonian University Medical College, ul. Mikołaja Kopernika 50, 31-501 Kraków, Poland

**Keywords:** giant basal cell carcinoma, giant cutaneous squamous cell carcinoma, Hedgehog pathway, PD-1 blockade, cemiplimab, tumour microenvironment, diagnostic delay, Mohs micrographic surgery, structured narrative review

## Abstract

Background/Objectives: Giant keratinocyte carcinomas, comprising basal cell carcinoma (BCC) and cutaneous squamous cell carcinoma (cSCC) with a maximum diameter ≥ 5 cm, pose substantial clinical challenges. This structured narrative review distinguishes giant-specific findings from contextual evidence in advanced or high-risk disease. Methods: The synthesis uses a retained bibliography assembled from searches described in earlier drafts as covering PubMed/MEDLINE and PubMed Central from January 2015 to March 2026, supplemented by citation and guideline searching and a focused update reported through 4 September 2026. A record-level search and screening log is unavailable; historical selection counts cannot be independently reconstructed. The review protocol was not registered, and exhaustive coverage or full PRISMA compliance is not claimed. Sources were classified by design and directness, with descriptive consideration of methodological limitations. Results: The retained set contains 51 sources: nine containing direct giant-specific clinical evidence and 42 providing contextual evidence. Direct evidence consists principally of case reports, retrospective or comparative studies and a giant-cSCC literature synthesis. It links giant BCC with prolonged lesion duration, ulceration, deeper invasion and reconstructive burden. Giant-cSCC outcomes vary substantially between reported series. Evidence for Hedgehog-pathway inhibition, PD-1 blockade and most molecular mechanisms derives mainly from conventional or advanced disease. Conclusions: Management requires multidisciplinary assessment integrating size, pathological risk, resectability and patient factors. Giant-specific molecular and therapeutic evidence remains limited, and contextual treatment effects should not be interpreted as effects established specifically in tumours ≥ 5 cm.

## Simple Summary

Basal cell carcinoma (BCC) and cutaneous squamous cell carcinoma (cSCC) are the most common keratinocyte malignancies. A small subset reaches an exceptionally large size, operationally defined in this review as a maximum diameter of ≥5 cm. These “giant” lesions may cause ulceration, bleeding, infection, pain, functional impairment and extensive tissue destruction, and they frequently require complex reconstruction, radiotherapy or systemic treatment. Direct evidence specific to tumours ≥5 cm is sparse. We therefore distinguish throughout between direct giant-tumour evidence and contextual evidence derived from high-risk, locally advanced or metastatic BCC/cSCC. We also discuss patient-, access- and health-system factors associated with delayed presentation while avoiding causal inference where only observational or indirect evidence is available.

## 1. Introduction

Keratinocyte carcinomas (KC)—basal cell carcinoma (BCC) and cutaneous squamous cell carcinoma (cSCC)—are the most common malignancies in fair-skinned populations and account for a substantial share of dermato-oncological activity, particularly in older adults [1,2,3,4,5,6]. Global Burden of Disease analyses document increasing BCC/cSCC burden over recent decades, with the highest age-standardised incidence rates reported in high-income North America and Australasia and particularly rapid increases in East Asia in recent analyses [6,7,8,9]. These population-level trends concern BCC/cSCC overall and do not demonstrate a parallel increase in the incidence of giant keratinocyte carcinomas.

The factors associated with such advanced presentation are multifactorial. Giant-tumour reports span case-based management discussions, clinical cohorts, comparative studies and a dedicated cSCC synthesis [10,11,12,13,14,15,16,17,18]. Direct giant basal cell carcinoma (GBCC) studies link larger tumour size with prolonged lesion duration and delayed presentation, and they describe psychosocial or social-context factors in selected cohorts [12,15,16]. The broader skin-cancer literature identifies additional barriers to timely assessment, including fear of disfigurement, comorbidity, limited access and health-system disruption [19,20,21,22,23,24,25,26,27]. These data support delay as an important correlate of advanced presentation without establishing a single causal pathway to the giant phenotype.

Giant keratinocyte carcinomas occupy a clinically important interface between tumour biology, host factors and delayed access to diagnosis or treatment. The revised evidence base includes sizeable direct GBCC cohorts and comparative histopathological studies [11,12,13,14,15,16], as well as giant-cSCC literature syntheses and a recent institutional series [17,18]. Nevertheless, direct evidence remains heterogeneous and predominantly retrospective; no prospective population-based cohort specifically designed around the ≥5 cm giant-KC phenotype was identified in the retained source set, and current data do not establish a unique size-dependent molecular phenotype.

This review brings together four domains: (i) the operational definition and clinicopathological context of giant keratinocyte carcinoma; (ii) anatomical, histopathological and staging features; (iii) molecular and therapeutic evidence; and (iv) patient-, access- and health-system factors associated with delayed presentation. Earlier giant-BCC management reports and giant-cSCC syntheses addressed selected parts of this question [10,17]. The specific aims are to summarise the directly observed clinical and pathological features of size-defined giant BCC/cSCC, distinguish them from evidence extrapolated from high-risk or locally advanced disease, critically describe the limitations of the available evidence and identify priorities for future size-specific research. The present synthesis therefore emphasises evidentiary directness rather than claiming exhaustive capture of the literature. Reports concerning facial-BCC differential diagnosis, melanoma/NMSC comorbidity and radiotherapy in general practice are used solely as contextual sources [28,29,30].

## 2. Materials and Methods

### 2.1. Review Design, Protocol and SANRA Framework

This manuscript is presented as a structured narrative review with narrative synthesis. During revision, the initial systematic-review framing was reconsidered because a record-level search and screening log is unavailable. This review therefore does not claim exhaustive database coverage, a reproducible historical selection process or full compliance with PRISMA 2020 [31]. No quantitative synthesis or meta-analysis was performed because tumour definitions, study designs, interventions and outcomes are heterogeneous.

The review protocol was not prospectively registered. Retrospective registration in PROSPERO or OSF after completion would not establish prospective specification and was therefore not undertaken. During the present revision, the six Scale for the Assessment of Narrative Review Articles (SANRA) domains—importance, aims, description of literature searching, referencing, scientific reasoning and presentation of relevant data—were used as a reporting-quality self-check for the narrative review. This retrospective use of SANRA is not presented as a prospectively prespecified protocol or as a formal risk-of-bias tool. Supplementary File S1 remains a retrospective methodological account. The unavailable historical search/screening log and lack of prospective registration limit reproducibility and assessment of selection bias.

### 2.2. Selection Criteria and Evidence Classification

The revised synthesis uses a modified Population–Exposure–Comparator–Outcome (PECO) framework and two principal evidence groups: direct giant-specific evidence and contextual evidence. Within the contextual group, studies of high-risk, locally advanced or metastatic disease are distinguished by source type and clinical domain from broader guidelines, reviews and the mechanistic or health-system literature:

Core Population/Evidence Stratum: Adults with histologically confirmed giant BCC or giant cSCC, operationally defined as a maximum tumour diameter ≥ 5 cm, at any anatomical site.

Contextual Evidence Group: Studies of high-risk, locally advanced or metastatic BCC/cSCC and guidelines or reviews that inform staging, molecular mechanisms, treatment, diagnostic delay or health-system organisation. This evidence is not used to generate giant-specific effect estimates.

Exposures/Comparators: Clinicopathological features, anatomical distribution, molecular alterations (including Hedgehog-pathway components, TP53/NOTCH/CDKN2A, PD-L1 expression and tumour mutational burden), treatment modality, and—where applicable—standard-risk KC or alternative treatment strategies.

Outcomes: Local control, recurrence, regional or distant metastasis, disease-specific and overall survival, treatment-related morbidity, reconstructive complexity, treatment response and toxicity, and diagnostic delay.

Primary reports containing size-defined giant BCC or cSCC contribute direct clinical evidence. Giant-specific secondary syntheses are classified as directly relevant to tumour size but are distinguished from primary data; their cases are not pooled with primary cohorts. In particular, the mixed-age giant-cSCC synthesis [17] cannot be interpreted as an exclusively adult cohort. Trials and cohorts without giant-size subgroup data, and broader reviews or guidelines, provide contextual evidence only. English- and Polish-language full texts were considered in the earlier search description; this restriction may have introduced language bias. The reported principal publication window was 1 January 2015 to 31 March 2026. The retained 2013 treatment review [32] is an explicitly identified contextual exception outside that window; it does not establish giant-specific treatment effects.

### 2.3. Search Strategy and Information Sources

Earlier manuscript versions described searches covering PubMed/MEDLINE and PubMed Central (PMC), supplemented by reference-list screening, citation tracking and guideline searching. PMC is an overlapping full-text archive, and unique retrievals cannot be attributed separately to it and PubMed/MEDLINE. The preceding revision reported a focused giant-specific update through 4 September 2026 and added eight reports [11,12,13,14,15,16,17,18], together with one reviewer-suggested health-system source [27]. Exact historical queries, execution dates and record-level retrieval and deduplication logs are unavailable. Consultations of Embase, Scopus, the Cochrane Library and Web of Science cannot be documented as reproducible systematic searches. Supplementary File S1 gives corrected reconstructions of the search concepts solely to explain the scope; these are not represented as verbatim historical queries or newly executed searches.

### 2.4. Retained Source Set and Selection-Accounting Limitations

**Figure 1:**
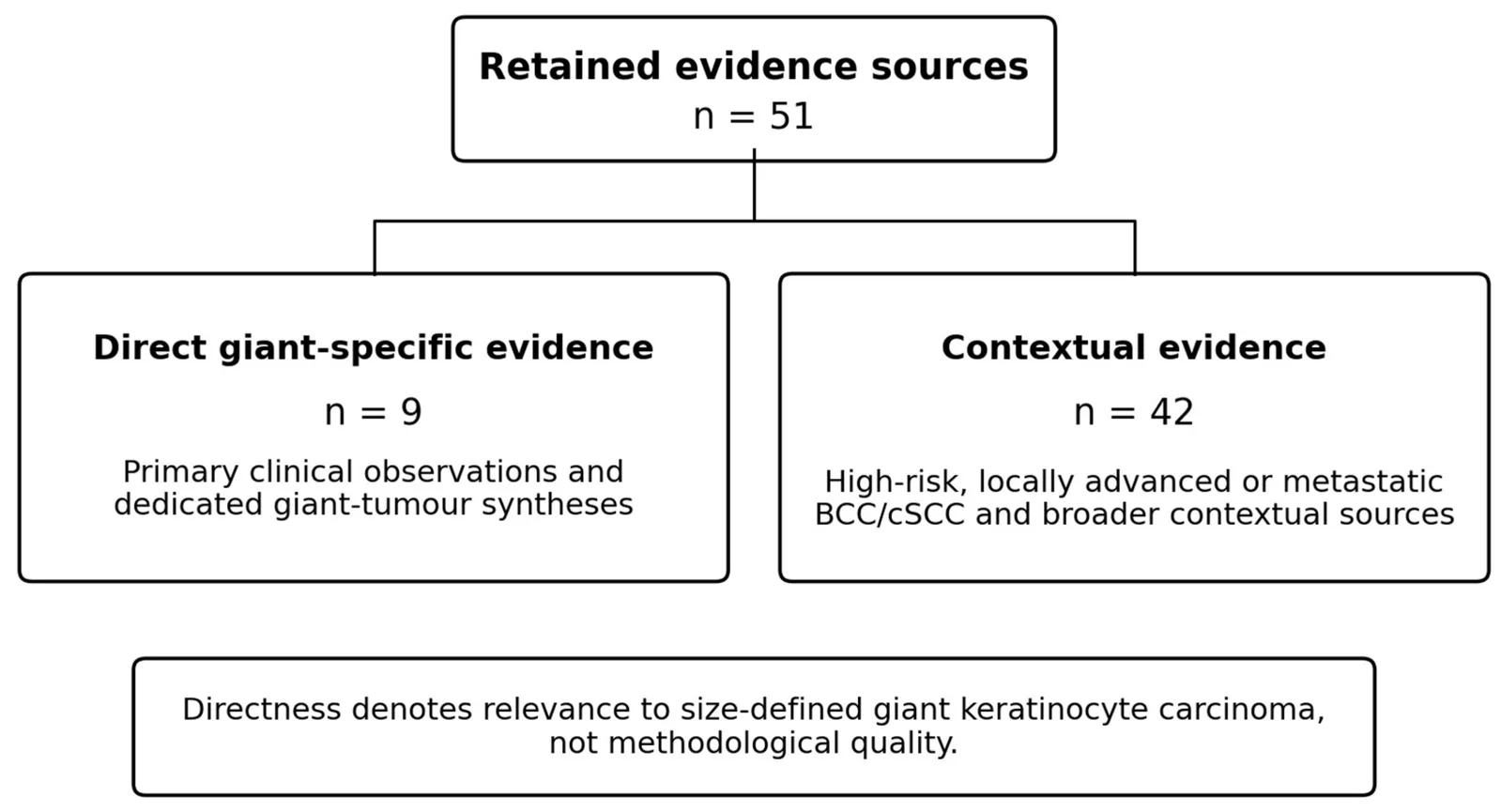
Composition of the 51 retained evidence sources. The figure describes the documented bibliography, not historical retrieval, screening or exclusion counts. Directness denotes relevance to size-defined giant keratinocyte carcinoma, not study quality. Mixed primary/secondary sources are counted once; the methods-only PRISMA reference is excluded. Direct giant-specific sources are references [10,11,12,13,14,15,16,17,18]. Source-level classifications are provided in Supplementary Tables S1 and S2.

The revised bibliography identifies 51 retained evidence sources, which are listed individually in Supplementary Table S1. The PRISMA reporting reference [31] is cited for methodological context and is not counted as a retained evidence source. Historical screening totals, exclusion categories and duplicate counts cannot be independently reconstructed from a record-level log and are therefore not presented as a verified selection flow. Figure 1 shows the composition of the retained source set rather than a PRISMA flow diagram. The absence of a screening log also prevents verification of the completeness of study capture and of the originally described independent screening process.

### 2.5. Data Extraction and Data Synthesis

The retained source summaries record bibliographic identity, design, disease/domain, size definition, available sample information, selected treatment/outcome information and evidentiary role. They are condensed source summaries rather than a complete duplicate-extraction dataset; country, anatomical detail, follow-up and numerical outcomes are not uniformly tabulated for every source. The earlier revision states that extraction was not fully independent and duplicated. No claim of independently verified duplicate extraction is made here. Sources were organised thematically, and statements based on advanced/high-risk rather than giant-size-defined populations are labelled contextual or extrapolative. No pooled patient count or pooled treatment effect was calculated, avoiding aggregation of potentially overlapping primary and secondary evidence.

### 2.6. Methodological Quality Assessment

The retained evidence spans case reports, observational studies, trials, evidence syntheses, clinical reviews and guidelines. Supplementary Table S2 describes design-related limitations, directness and the role of each source in the narrative. The relevant considerations overlap with domains addressed by JBI, AMSTAR-2 and AGREE-II; however, completed item-level assessments and supporting judgements are not reported in this package. The table must therefore not be interpreted as a completed formal assessment using those instruments. No numerical quality scores, overall certainty grades or formal quality-based weights were assigned. This descriptive appraisal is a limitation of this review, not a substitute for a full risk-of-bias assessment.

### 2.7. Directness and Interpretive Safeguards

Direct giant-specific evidence includes a source when it contains primary clinical observations or a dedicated secondary synthesis of size-defined giant tumours. Mott et al. [10] is counted as direct for its giant-BCC case, while its general management discussion remains contextual. The giant-cSCC synthesis [17] is directly relevant to size but remains secondary and includes mixed ages. All other sources without giant-size clinical data are contextual. Directness is distinct from methodological quality: a directly relevant case report cannot establish comparative treatment efficacy, whereas an advanced-disease trial may inform treatment despite uncertain transferability to giant tumours. Contextual statements are not converted into giant-specific effect estimates. No GRADE assessment was performed.

## 3. Composition and Characteristics of the Retained Evidence

### 3.1. Retained Source Set

Supplementary Tables S1 and S2 contain the same 51 evidence sources. Nine sources contain direct giant-specific evidence [10,11,12,13,14,15,16,17,18]; the remaining 42 provide contextual evidence. This source-level classification is shown in Figure 1. These counts describe the final bibliography and do not reconstruct historical screening decisions.

### 3.2. Evidence Composition and Directness

Direct evidence includes a giant-BCC case with management discussion [10], surgical series, comparative histopathology or case–control studies and retrospective GBCC cohorts [11,12,13,14,15,16]; a giant-cSCC literature synthesis [17]; and a giant-cSCC institutional series [18]. The retained summaries describe the original size thresholds rather than treating ≥5 cm and >5 cm as identical eligibility rules. The giant-cSCC literature synthesis includes mixed ages and historical staging systems; its aggregate results are not adult-only estimates. Evidence for systemic therapy, most molecular mechanisms and service-delivery factors predominantly comes from contextual studies. Supplementary Table S1 provides concise source characteristics and explicitly identifies this difference in evidentiary role.

### 3.3. Methodological Appraisal

The direct GBCC cohorts and comparative studies [11,12,13,14,15,16] and giant-cSCC reports [17,18] are more closely aligned with the size-defined question than advanced-disease trials but remain susceptible to selection, referral and reporting bias. Prospective trials provide more informative therapeutic evidence in their enrolled advanced-disease populations, but their results do not establish effects in a separate giant-size subgroup. Supplementary Table S2 records descriptive limitations and intended use in the synthesis without formal tool scores or certainty grades.

## 4. Definition and Classification of Giant Keratinocyte Carcinomas

For the purposes of this review, “giant” keratinocyte carcinoma is operationally defined as a BCC or cSCC with a maximum diameter ≥5 cm. This threshold is widely used in giant-BCC series and comparative studies [11,12,13,14,15,16] and has also informed published giant-cSCC syntheses [17,18], but it should be regarded as a pragmatic harmonisation rule rather than a universally validated biological boundary. Some studies use “>5 cm” rather than “≥5 cm”, and the older literature has used alternative thresholds. Tumour size should therefore be interpreted alongside depth, anatomical invasion, histopathological risk features and resectability.

Size alone is insufficient to capture biological behaviour and therapeutic complexity. Current frameworks emphasise ulceration, depth of invasion (including extension beyond subcutaneous tissue), perineural invasion (PNI), bone involvement, lymphovascular invasion (LVI), poor differentiation and host-related factors such as immunosuppression as independent determinants of recurrence, metastasis and disease-specific outcomes [2,3,5,33,34]. In cSCC, current guidance recommends multiparameter risk stratification integrating clinical findings, histopathology and validated staging systems—primarily the American Joint Committee on Cancer (AJCC) 8th edition and the Brigham and Women’s Hospital (BWH) tumour classification—rather than reliance on a single dimensional cut-off [2,3,33,34,35,36,37]. In BCC, the concept of “difficult-to-treat” or “locally advanced” disease incorporates location (H-zone of the face, periorbital region, and scalp), recurrence after prior therapy, histological subtype (e.g., infiltrative, basosquamous) and feasibility of radical surgery without unacceptable morbidity [1,4,5].

Direct GBCC studies consistently show that very large lesions are more often ulcerated, thicker and/or infiltrative than conventional BCC, although these features may partly reflect accumulated tumour size and duration rather than a distinct biological programme [12,13,14,15,16]. Giant cSCC similarly requires interpretation through established adverse features—depth, poor differentiation, perineural invasion, lymphovascular invasion and host immunosuppression—rather than diameter alone [2,3,17,18,33,34]. BCC remains predominantly locally destructive, whereas cSCC has greater metastatic potential when additional high-risk features are present.

Beyond anatomical and pathological criteria, the EADO/EDF and NCCN frameworks recognise functional dimensions of advanced disease: the inability to achieve a curative-intent surgical resection without unacceptable functional or cosmetic morbidity and persistence or recurrence despite optimal locoregional therapy. These criteria explicitly bring locally advanced BCC and high-risk cSCC into the domain of systemic therapy and multidisciplinary tumour board discussion [1,2,3,4,5].

## 5. Epidemiology and Anatomical Distribution

Giant tumours constitute a small minority of keratinocyte carcinomas. In direct GBCC cohorts, reported proportions vary by setting: Approximately 0.5–1% is commonly cited, while one large retrospective series identified 115 GBCCs among 5958 BCC patients (2%) [14]. These are institution- or referral-based proportions rather than population prevalence estimates. Direct studies consistently report older populations and frequent head-and-neck involvement, although site distributions differ among cohorts [11,14,15,16]. Giant-cSCC series likewise show a predominance of head/scalp/face sites, but no robust population-based incidence or prevalence estimate for giant cSCC was identified [17,18].

At the population level, global analyses document a substantial increase in the absolute burden of non-melanoma skin cancer over recent decades, particularly in older adults [6,7,8,9]. These studies support a growing population-level burden but should not be interpreted as providing a direct estimate of the prevalence of the giant phenotype. Even if giant lesions remain uncommon, demographic ageing and the increasing overall number of keratinocyte carcinomas make continued demand for multidisciplinary oncology, reconstructive surgery, radiotherapy and systemic therapy plausible; this is a health-service inference rather than a size-specific incidence projection.

Direct GBCC studies associate larger tumours with prolonged disease duration/delay and describe social or behavioural correlates in selected cohorts [12,15,16]. In the 43-patient Oudit cohort, tumour size correlated with delay in presentation [16]. Immunosuppression—particularly after solid-organ transplantation, but also in haematological disease, HIV infection and iatrogenic immunosuppression—is an established risk factor for aggressive cSCC overall [2,3,5,33,38]; however, a giant-tumour-specific relative-risk estimate cannot be derived from the present evidence.

## 6. Histopathology, Staging and Predictors of Aggressive Course

### 6.1. Cutaneous Squamous Cell Carcinoma

High-risk histological features in cSCC include invasion beyond subcutaneous fat, poor differentiation, lymphovascular invasion (LVI) and perineural invasion (PNI). PNI definitions are framework-dependent: AJCC8 upstaging considers tumour involvement of nerves ≥ 0.1 mm in calibre, nerves deeper than the dermis, or named nerves without skull-base invasion, whereas BWH staging treats PNI of nerves ≥ 0.1 mm as one of its independent high-risk factors; NCCN risk stratification additionally distinguishes clinical/radiographic PNI and large-calibre or deep nerve involvement [2,3,5,32,33,34,35,36]. Depth should be reported as tumour thickness in millimetres and as the deepest anatomic level of invasion (dermis, subcutaneous fat, fascia, muscle or bone), because these dimensions have different staging implications. These factors correlate with local recurrence and regional or distant metastasis and inform decisions regarding margin control, nodal evaluation, imaging and adjuvant therapy.

Contemporary data validate NCCN high-risk and very-high-risk groupings for prognostic enrichment and treatment planning [37]. In the validation cohort reported by Stevens and colleagues, very-high-risk classification was associated with increased local recurrence, nodal metastasis and disease-specific death [37]. For head-and-neck cSCC, a tumour ≥ 5 cm fulfils at least the AJCC8 T3 size criterion; invasion of specified structures may justify T4 classification, whereas BWH staging cannot be inferred from diameter alone because it depends on additional independent risk factors. The direct giant-cSCC literature confirms substantial heterogeneity: the 2021 synthesis reported low absolute rates of nodal/distant metastasis across published cases, whereas a recent seven-patient institutional series reported nodal metastases in 42.9% [17,18]. These differences underline the influence of case selection and additional pathological risk features.

Histological subtype carries molecular implications. Desmoplastic cSCC is characterised by stromal collagen remodelling and infiltrative single-cell or small-cluster invasion and is associated with higher rates of PNI and local recurrence. Acantholytic and adenosquamous variants have also been linked with more aggressive behaviour, although these associations vary across series. Standardised synoptic reporting—including tumour thickness in millimetres’ depth of invasion relative to subcutaneous fat, fascia, muscle and bone, differentiation grade, PNI status with nerve calibre, LVI status and margin distance—should be regarded as the minimum dataset for any high-risk or giant cSCC [33,34,35,36,37].

### 6.2. Basal Cell Carcinoma

In BCC, aggressive histological subtypes—infiltrative/morpheaform, micronodular and basosquamous variants—together with critical-site locations increase the risk of local recurrence and often justify micrographically controlled surgery or multidisciplinary discussion [1,4,5]. Direct comparative GBCC studies report greater tumour thickness, ulceration and infiltrative growth than conventional BCC, with high-grade histological variants enriched in some cohorts [12,13,14,15]. In the 43-patient Oudit cohort, only two patients (<5%) presented with extensive local invasion; one also had distant metastases, and both died of disease, whereas surgically treated patients otherwise had favourable local-control outcomes [16]. This giant-BCC-specific series supports the interpretation that metastatic behaviour remains uncommon even among very large BCCs, although deeply invasive or biologically aggressive cases can be lethal.

In addition to subtype, clinical features such as H-zone involvement, ulceration extending to fascia or bone, recurrence after prior therapy and incomplete prior excision are associated with treatment complexity and adverse outcomes and underpin the “difficult-to-treat” designation [1,4,5]. Basosquamous carcinoma remains a distinct histopathological entity within the keratinocyte-carcinoma spectrum; its overlapping clinical aggressiveness with cSCC should not be interpreted as loss of diagnostic distinction. Accurate histopathological classification is particularly important because basosquamous and deeply invasive giant BCC may carry a higher metastatic risk than conventional BCC.

**Figure 2:**
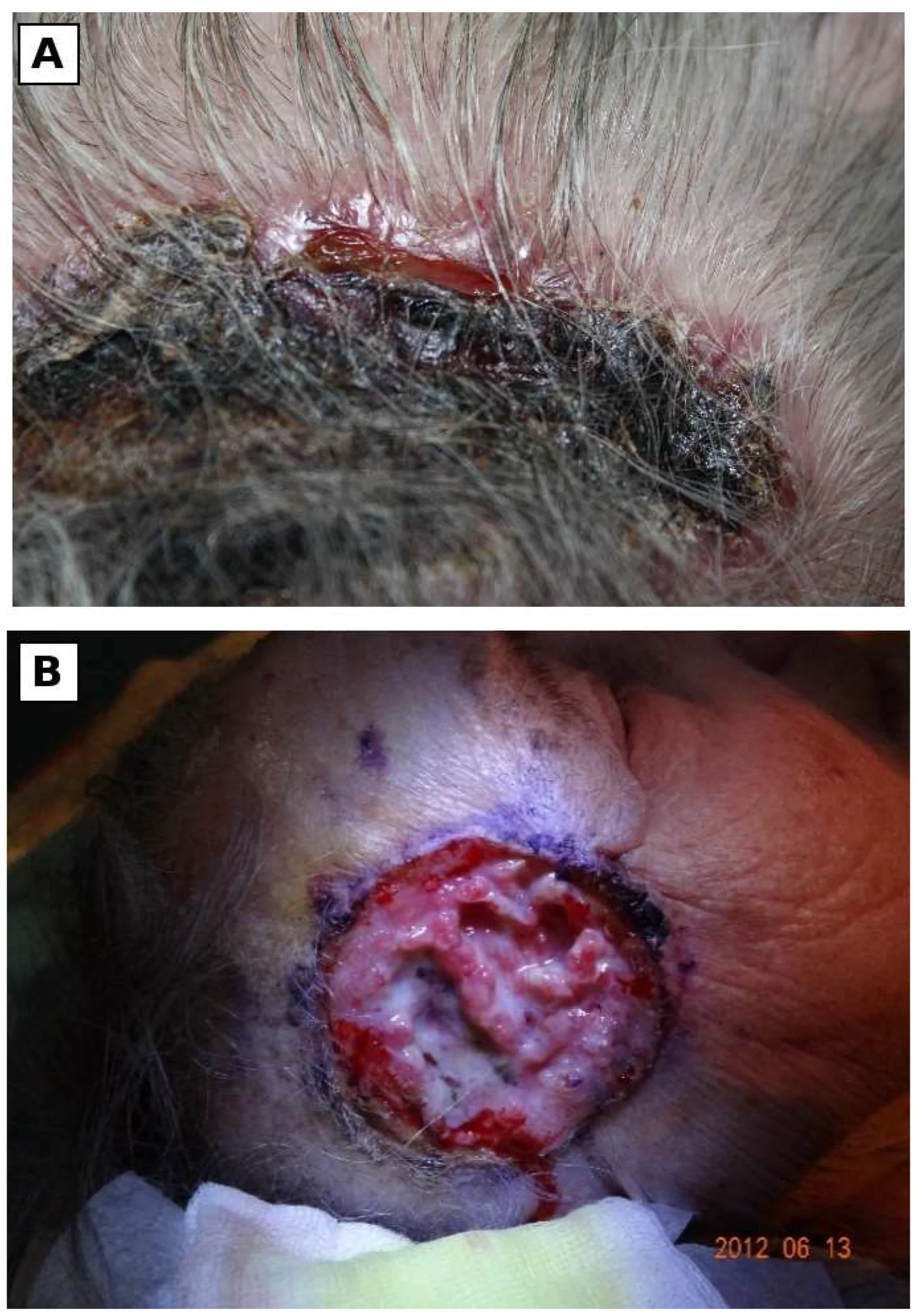
Clinical photographs of ulcerated basal cell carcinoma, reproduced from the original manuscript. (**A**) Crusted and ulcerated lesion on the hair-bearing scalp. (**B**) Ulcerated lesion shown in the second original photograph. The panels are illustrative; no shared patient identity, treatment sequence or measured tumour diameter is inferred from these photographs. Written informed consent for publication was obtained.

Illustrative clinical photographs of ulcerated BCC are shown in Figure 2. They are not included in the retained literature-source counts or used to establish tumour size or treatment outcomes.

## 7. Molecular Evidence with Clinical Translation: Hedgehog, PD-1 and Extracellular Matrix

### 7.1. Hedgehog Signalling in BCC and Resistance

Contextual evidence from conventional and advanced BCC establishes constitutive Hedgehog (Hh) pathway activation—most commonly through PTCH1 loss-of-function or SMO activation—as a central mechanism of BCC pathogenesis [39]. Comparative translational data in giant BCC are limited and do not establish a validated giant-specific Hedgehog-pathway signature; therefore, the pathway biology summarised here should be interpreted as mechanistically relevant to BCC generally rather than as a demonstrated giant-specific signature. Hh activation promotes GLI1/GLI2-dependent transcription regulating proliferation, survival and stemness.

Hedgehog inhibitors are limited by primary and acquired resistance and by a characteristic toxicity profile—muscle spasms, dysgeusia, alopecia, weight loss and amenorrhoea—which together compromise long-term adherence in a substantial proportion of patients [39,40,41]. Strategies described in the literature include switching between SMO inhibitors, intermittent dosing schedules, integration with local modalities (radiotherapy and surgery) and sequencing immunotherapy after disease progression or intolerance [39,40,41]. Mechanisms of acquired resistance include secondary mutations in SMO altering the drug-binding pocket, GLI amplification, activation of bypass signalling pathways (PI3K/mTOR, RAS/MAPK) and stem-cell-like reprogramming within the tumour compartment [39,40]. A recent meta-analysis confirmed meaningful activity of Hedgehog inhibitors in advanced BCC, with variable effectiveness across locally advanced versus metastatic settings and an important rate of treatment discontinuation due to adverse events [41].

### 7.2. PD-1 Blockade in Advanced cSCC and BCC

Immune checkpoint inhibition has reshaped the management of advanced cSCC. UV-associated cSCC has a high tumour mutational burden, broad neoantigen repertoire and an immunogenic tumour microenvironment that provide a strong biological rationale for PD-1 blockade [42,43,44,45]. Cemiplimab has demonstrated clinically meaningful and durable activity in metastatic and locally advanced cSCC across pivotal trials and prospective real-world cohorts [42,43,46]. Pembrolizumab has also demonstrated durable activity in KEYNOTE-629 and is an established anti-PD-1 option in appropriate advanced-disease settings [47]. These treatment data derive predominantly from advanced/high-risk cSCC rather than cohorts selected by the ≥5 cm giant-tumour threshold and should therefore be interpreted as contextual therapeutic evidence.

In BCC, PD-1 blockade is most relevant after Hedgehog-inhibitor failure or intolerance. A phase 2 trial of cemiplimab in locally advanced BCC after Hedgehog-inhibitor therapy demonstrated clinically meaningful antitumour activity with acceptable safety, supporting treatment sequencing after pathway-directed therapy [48,49]. The mechanistic rationale includes an immunologically active microenvironment in a subset of advanced BCCs [44,49]. Neoadjuvant PD-1 blockade has also shown substantial pathological activity in resectable high-risk cSCC [50]; however, this evidence should not be interpreted as giant-tumour-specific unless patients meeting the ≥5 cm definition are analysed separately.

### 7.3. ECM Remodelling and the Tumour Microenvironment

The tumour microenvironment and the extracellular matrix (ECM) contribute to invasion, immune evasion and therapy response in both BCC and cSCC. Recent syntheses highlight stromal compartment remodelling—activation of cancer-associated fibroblasts (CAFs), dynamic changes in collagen organisation (from a basket-weave to a parallel-aligned, tumour-permissive architecture) and altered hyaluronan, laminin and proteoglycan deposition—and their interaction with inflammatory cytokine signalling and immune checkpoint pathways [45,51]. Matrix metalloproteinases (MMP-1, MMP-2 and MMP-9), lysyl oxidase (LOX) and tissue inhibitors of metalloproteinases (TIMPs) participate in this remodelling and have been implicated in cSCC invasiveness and the establishment of a fibrotic, immunosuppressive niche [45,51].

**Figure 3:**
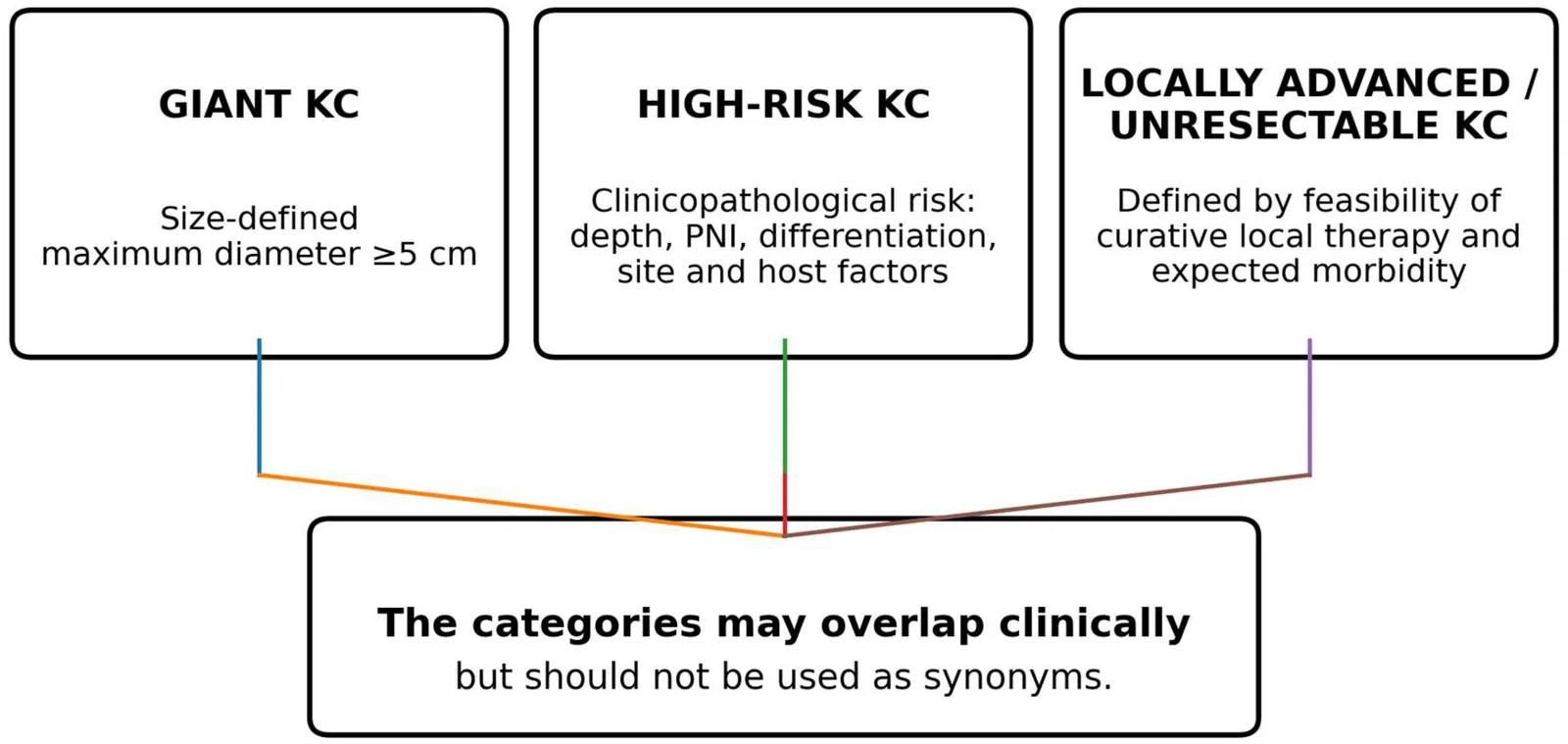
Conceptual distinction between size-defined giant keratinocyte carcinoma, clinicopathologically high-risk disease and locally advanced/unresectable disease. The categories may overlap but should not be used as synonyms.

In giant tumours, hypoxia, chronic inflammation, ulceration and secondary infection are common clinical features that may plausibly interact with ECM remodelling and immune regulation. However, the retained giant-specific studies do not establish a distinct ECM signature, nor do they validate MMPs, LOX, TIMPs, stromal alignment or related microenvironmental features as size-specific biomarkers. Accordingly, the ECM mechanisms summarised above are extrapolated from the conventional or advanced BCC/cSCC tumour-microenvironment literature [45,51] and should be treated as hypotheses requiring direct comparison in size-defined cohorts. Size-specific translational datasets remain a research priority. A comparative synthesis of giant BCC and giant cSCC, with contextual molecular and therapeutic implications, is provided in Table 1. Table 2 and Figure 3 summarise the distinction between size-defined giant tumours, high-risk disease and locally advanced or unresectable disease.

## 8. Therapeutic Strategies in Giant Keratinocyte Carcinoma: Surgery, Radiotherapy and Systemic Therapy

### 8.1. Surgery and Margin Control

Complete surgical excision remains the first-line treatment for most resectable BCC and cSCC, with margin strategy tailored to risk and anatomical constraints [1,2,3,4,5]. In giant lesions, fixed numerical margins derived from standard-risk disease should not be applied uncritically; margin control, resectability and reconstructive consequences should be planned individually, preferably in a multidisciplinary setting. Micrographic surgery is appropriate only when technically feasible and oncologically suitable for complete margin assessment. Very large tumours with extensive deep soft-tissue, bone or major neurovascular involvement may exceed the practical or oncological limits of Mohs micrographic surgery and instead require staged/wide resection, reconstruction and/or non-surgical modalities.

For high-risk head-and-neck cSCC, observational comparative data indicate a lower recurrence rate after Mohs micrographic surgery than after standard excision, but the evidence is retrospective rather than randomised [52]. Therefore, the choice between Mohs surgery, staged excision and conventional wide excision in giant tumours should be individualised according to site, depth, resectability, required reconstruction, availability of complete margin assessment and patient factors. Multidisciplinary planning involving dermatologic surgery, head-and-neck or plastic reconstruction, radiation oncology and medical oncology is particularly important for lesions involving bone, deep fascia or major neurovascular structures [1,2,3,4,5].

### 8.2. Radiotherapy

Radiotherapy is used as definitive therapy when surgery is contraindicated by patient comorbidity, anatomical constraints or patient preference and as adjuvant therapy in high-risk cSCC or BCC—for example, in PNI involving named or large-calibre nerves, close or positive margins where re-excision is not feasible, and multiply recurrent tumours. Definitive radiotherapy doses for advanced keratinocyte carcinoma typically range from 60 to 70 Gy in conventional fractionation, with hypofractionated regimens used in elderly and frail patients; adjuvant radiotherapy is generally delivered at 50 to 60 Gy [1,2,3,5]. Modern techniques (intensity-modulated radiotherapy, volumetric modulated arc therapy, image-guided radiotherapy and electron-beam therapy for superficial sites) allow conformal dose delivery with relative sparing of organs at risk. Large ulcerated tumours and complex wound beds may complicate planning, increase the risk of acute mucocutaneous toxicity and chronic radionecrosis and require multidisciplinary assessment of the trade-off between disease control and functional preservation [1,2,3,5].

### 8.3. Systemic Therapy and Treatment Sequencing

For advanced cSCC, anti-PD-1 inhibitors are established systemic options, including cemiplimab and pembrolizumab; dosing and scheduling should follow current product labelling and applicable oncology guidance rather than be inferred from tumour size alone [2,3,42,43,46,47]. By contrast, neoadjuvant PD-1 blockade in resectable high-risk cSCC is an emerging strategy supported by prospective trial data [50] and should not be conflated with established treatment specifically for giant cSCC. These data are highly relevant to multidisciplinary decision-making in giant cSCC but remain extrapolative unless a ≥5 cm subgroup is separately reported.

For advanced BCC, Hedgehog-pathway inhibitors remain foundational systemic therapy, with anti-PD-1 therapy used after Hedgehog-inhibitor failure or intolerance in appropriate patients [1,4,39,40,41,48,49]. Treatment sequencing may include pathway inhibition followed by immunotherapy and integration with locoregional therapy, but evidence specific to tumours ≥5 cm remains limited. Systemic therapy is most relevant for unresectable disease, disease in which surgery would cause unacceptable functional or cosmetic morbidity, multifocal disease or metastatic disease.

### 8.4. Multidisciplinary Care, Supportive Therapy and Rehabilitation

Patients with giant keratinocyte carcinoma frequently present with complex needs that extend beyond oncological treatment. Active management of ulceration, malodour, pain and bleeding from large tumours—including topical antiseptics, advanced wound dressings, nutritional optimisation and assessment and treatment of secondary infection when clinically indicated—is essential to maintain quality of life and treatment tolerance. Pre- and postoperative rehabilitation, lymphoedema management, speech and swallowing therapy in head-and-neck cases and psychological support addressing fear of disfigurement and post-treatment body image are integral components of contemporary care [1,2,3,4,5,32]. Comprehensive geriatric assessment is recommended in elderly patients to align treatment intensity with overall health status and patient preferences.

## 9. Patient-, Access- and Health-System Factors Associated with Delayed Presentation and Diagnosis

Delayed presentation of giant keratinocyte carcinoma is typically multifactorial. A working taxonomy distinguishes patient-level, healthcare-access, diagnostic-pathway and system-shock contributors:

- Patient-Level Factors: Low cancer literacy; normalisation of chronic ulceration or dermatoses; denial and fear of cancer diagnosis; fear of facial surgery and disfigurement; mobility limitations; cognitive decline; social isolation; competing health priorities in patients with multimorbidity [19,20,21]. The melanoma-focused delay study [21] provides indirect context and does not establish the frequency or causes of delay in giant BCC/cSCC.
- Healthcare-Access Factors: Limited dermatology capacity, particularly in rural and underserved regions; referral bottlenecks from primary care; absence of integrated skin cancer pathways with explicit timelines; underfunding of multidisciplinary clinics; geographic and economic barriers to specialist care [5,21,32].

Diagnostic Pathway Performance: Teledermatology can mitigate access barriers, particularly for triage, but its diagnostic performance depends on image quality, modality, case mix and reference standard. In a systematic review and meta-analysis, pooled overall diagnostic agreement was approximately 68.9%, while agreement between teledermatology and histopathology was lower (approximately 55.7%) [23]. Teledermatology should therefore be implemented with quality-assurance safeguards, dermoscopic image standards where appropriate and explicit escalation pathways for biopsy or face-to-face assessment.

System Shocks (COVID-19 and Beyond): Pandemic-related disruption was associated with marked reductions in skin-cancer diagnostic and surgical activity. A 2024 systematic review and meta-analysis including melanoma and keratinocyte carcinomas reported a substantial early-pandemic reduction in keratinocyte-carcinoma surgery and evidence of greater tumour thickness or stage in pandemic-era cases [25]. Institutional and case–control datasets similarly documented delayed diagnosis or treatment [22,24,26]. These findings support health-system disruption as a contributor to delayed presentation, although they do not establish a direct causal pathway to the giant phenotype in individual patients.

Gentileschi et al. described the reduction in elective surgical activity during the early pandemic and argued for prioritising selected aggressive or rapidly growing skin cancers, including BCC and cSCC [27]. Their discussion links postponement with the possibility of deeper invasion, greater reconstructive requirements and functional impairment. This is a health-system and clinical context; it does not measure an increase in the incidence of giant tumours. The distinction matters because plausible mechanisms of delay cannot substitute for direct population evidence.

## 10. Discussion

The principal contribution of this synthesis is the explicit separation of what has been observed in size-defined giant tumours from what is inferred from broader keratinocyte-carcinoma evidence. Direct studies inform presentation, histopathology, delay and reconstructive burden. Advanced-disease trials inform systemic-treatment selection but do not establish a giant-specific treatment effect. Similarly, plausible molecular and health-system mechanisms remain hypotheses about the giant phenotype unless tested in size-defined populations. The available evidence is most consistent with a multifactorial model in which prolonged diagnostic/treatment delay and adverse tumour biology may both contribute to extreme tumour size; the relative contribution of each factor cannot be quantified from the present literature.

## 11. Strengths and Limitations of This Study

A strength of this review is the deliberate source-level separation of direct giant-specific evidence from contextual evidence in high-risk, locally advanced or metastatic disease, which reduces the risk of presenting advanced-disease treatment or molecular data as if they were size-specific. This review also integrates clinicopathological, therapeutic and health-system dimensions and preserves original size thresholds rather than pooling heterogeneous definitions.

Several limitations constrain inference. The historical record-level search and screening log is unavailable, so search completeness, duplicate handling, screening decisions and exclusion totals cannot be independently reconstructed. The publication windows and update dates are reported from earlier manuscript descriptions rather than verified execution logs. This review is therefore a structured narrative synthesis, not an exhaustive systematic review. There is no prospectively registered protocol; the original extraction was not fully duplicated; the condensed source summaries do not supply complete item-level formal risk-of-bias assessments. The nine direct sources are heterogeneous, include mixed primary/secondary reports and a mixed-age giant-cSCC synthesis, and may overlap in secondary case material; patient counts have therefore not been pooled. Case selection, inconsistent use of >5 cm versus ≥5 cm, incomplete follow-up, and restriction to English/Polish full text may introduce selection and language bias. Most therapeutic and molecular evidence remains contextual.

## 12. Future Directions

Priority studies should use prospective multicentre or registry-based designs with explicit size measurement, harmonised reporting of depth and staging, and longitudinal outcome capture. Size-stratified translational studies should test whether Hedgehog-pathway activity, PD-L1 expression, tumour mutational burden, ECM-remodelling markers or immune-cell phenotypes differ independently with giant size rather than merely with stage, ulceration or treatment history. These variables should currently be regarded as candidate biomarkers requiring prospective validation, not established predictors of the giant phenotype. Clinical studies should also report whether giant lesions are represented in neoadjuvant or systemic-therapy cohorts and should prospectively evaluate whether expedited referral, teledermatology pathways or surveillance of high-risk patients reduces late presentation.

## 13. Conclusions

Giant KC is a size-defined presentation (operationally ≥ 5 cm) and should not be treated as synonymous with conventional high-risk, locally advanced or unresectable BCC/cSCC. Compared with conventional BCC/cSCC, giant tumours carry greater local tissue destruction and reconstructive burden because of their size and duration; however, biological aggressiveness is not uniform. Giant BCC remains predominantly locally destructive with uncommon metastasis, whereas giant cSCC may carry substantial nodal risk when additional adverse pathological features are present. Management should therefore integrate size with depth, histological risk features, anatomical involvement, resectability, host factors and patient goals. Surgery remains the principal curative modality when feasible; radiotherapy and systemic therapy should follow established risk- and stage-based indications. Hedgehog-pathway and PD-1 data are clinically relevant but largely extrapolated from advanced-disease populations rather than proven specifically in ≥5 cm tumours. Prospective size-specific cohorts are required before molecular markers, treatment effects or health-system interventions can be considered validated for the giant phenotype.

## Figures and Tables

**Table 1:** Comparative clinical characteristics of giant basal cell carcinoma (BCC) and giant cutaneous squamous cell carcinoma (cSCC), with explicitly contextual molecular and therapeutic evidence.

Category	Giant BCC	Giant cSCC	Contextual Mechanisms and Implications
Operational definition	Operational threshold of ≥5 cm; some reports use >5 cm. Locally advanced disease additionally depends on anatomy, prior treatment and resectability [1,4,10,11,12,13,14,15,16].	Operational threshold of ≥5 cm; size is interpreted with histopathology, anatomical invasion and AJCC-8/BWH risk criteria [2,3,17,18,33,34,35,36,37].	Tumour size is not a validated molecular boundary. Giant-specific pathway and biomarker data remain limited.
Main risk factors	Chronic UV exposure; fair phototype; advanced age; male sex; immunosuppression; genetic syndromes (e.g., Gorlin); delayed diagnosis [1,4,5].	Chronic UV exposure; actinic keratosis/field cancerisation; chronic scars; immunosuppression (e.g., transplantation); delayed diagnosis/access barriers [2,3,5,33,38].	UV-related DNA damage and immune context are relevant to both cancers; Hedgehog-pathway activation is central to BCC. These mechanisms are not established as unique to giant tumours.
Histopathology (giant lesions)	Direct studies report ulceration, greater thickness and infiltrative growth; enrichment of aggressive subtypes varies across cohorts [12,13,14,15,16].	Size-defined reports are heterogeneous. Depth, differentiation, PNI and nodal status remain clinically relevant; giant-specific prognosis is not determined by size alone [17,18,33,34].	BCC: aberrant Hh signalling (PTCH1/SMO/GLI). cSCC: TP53/NOTCH/CDKN2A alterations; EMT programmes; immune escape signatures in advanced disease.
Clinical behaviour (evidence strength varies)	Local destruction and reconstructive burden predominate; metastatic and fatal outcomes can occur in selected giant-BCC reports [10,11,12,13,14,15,16].	Nodal and distant outcomes vary across giant-cSCC series and are influenced by case selection and pathological features [17,18].	cSCC: high neoantigen load → sensitivity to PD-1 blockade. BCC: immunotherapy best established after HHI failure or intolerance.
Staging/risk stratification	Resectability + high-risk features; concept of locally advanced BCC [1,4,5].	AJCC-8/BWH + NCCN/EADO high-risk criteria [2,3,5,35,36,37].	Molecular markers not yet standard in staging; ongoing work on genomic and immune classifiers.
Local treatment	Complete wide/staged excision with reconstruction is reported in a direct series. Micrographically controlled surgery is selected according to guideline-based risk and feasibility [1,4,10,11,16].	Radical excision with risk-adapted margins; nodal evaluation; adjuvant radiotherapy in high-risk contexts [2,3,5].	Molecular targets guide systemic therapy rather than margin strategy.
Systemic therapy	Locally advanced or unresectable: HHI; anti–PD-1 after HHI failure or intolerance [1,4,39,40,41,48,49].	Advanced/unresectable disease: anti–PD-1 therapy (cemiplimab or pembrolizumab) according to current guidance and patient/tumour factors [2,3,42,43,46,47,50].	Hedgehog inhibition (BCC); PD-1 axis blockade (cSCC) linked to high TMB/neoantigens and TME context.
Prognosis	Outcome varies with local invasion, resectability and case selection. Large size alone does not establish metastatic behaviour [11,12,13,14,15,16].	Stage- and setting-dependent; giant-cSCC reports show heterogeneous metastatic and survival outcomes [17,18].	No validated giant-specific molecular prognostic classifier is established in this review.

Sources: guideline-based management and staging [1,2,3,4,5,33,34,35,36,37]; giant-specific clinical reports [10,11,12,13,14,15,16,17,18]; advanced-disease treatment and tumour-microenvironment literature [39,40,41,42,43,44,45,46,47,48,49,50,51,52]. Table 1 is a clinical summary, not a set of giant-specific comparative effect estimates. Directness and design-related limitations are listed source by source in Supplementary Tables S1 and S2.

**Table 2:** Conceptual distinction between giant, high-risk and locally advanced/unresectable keratinocyte carcinoma.

Feature	Giant KC	High-Risk KC	Locally Advanced/Unresectable KC
Defining principle	Maximum diameter ≥ 5 cm (operational definition in this review)	Clinicopathological features associated with recurrence/metastasis	Feasibility of curative local therapy and expected morbidity
Relationship to size	Size-defined by definition	May be small or large	May be small or large
Biological implication	Large size alone does not prove a unique molecular phenotype	Risk reflects depth, PNI, differentiation, site, host factors and other criteria	Advanced status reflects anatomy, prior treatment, invasion and resectability; biology may vary
Evidence used in this review	Direct size-defined reports [10,11,12,13,14,15,16,17,18]	Guidelines and high-risk cohorts [1,2,3,4,5,33,34,35,36,37]	Advanced-disease trials/reviews [20,39,40,41,42,43,44,45,46,47,48,49,50]
Therapeutic inference	Individualised; surgery if feasible, with reconstruction as needed	Risk-adapted local/regional management	Systemic therapy and/or radiotherapy when curative surgery is not feasible or acceptable

## Data Availability

No new analytical patient-level dataset was generated. Figure 2 reproduces illustrative clinical photographs from the original manuscript. The retained source list and descriptive summaries are provided in Supplementary Tables S1 and S2. Supplementary File S1 contains a retrospective methodological account. A historical record-level search and screening log is unavailable.

## Supplementary Materials

The following supporting information can be downloaded at https://www.mdpi.com/article/10.3390/cancers18203367/s1. Supplementary File S1: retrospective methodological account and reconstructed search concepts. Supplementary Table S1: concise characteristics and evidentiary roles of all 51 retained sources. Supplementary Table S2: descriptive methodological limitations, directness and use of each source in the narrative synthesis.

## Abbreviations

The following abbreviations are used in this manuscript:

AGREE-II	Appraisal of Guidelines for Research and Evaluation, version II
AJCC-8	American Joint Committee on Cancer, 8th edition
AMSTAR-2	A Measurement Tool to Assess Systematic Reviews, version 2
BCC	Basal cell carcinoma
BWH	Brigham and Women’s Hospital tumour staging for cSCC
CAF	Cancer-associated fibroblast
CDKN2A	Cyclin-dependent kinase inhibitor 2A
cSCC	Cutaneous squamous cell carcinoma
DSS	Disease-specific survival
EADO	European Association of Dermato-Oncology
ECM	Extracellular matrix
EDF	European Dermatology Forum
EMT	Epithelial–mesenchymal transition
GBD	Global Burden of Disease
GLI1/GLI2	Glioma-associated oncogene transcription factors 1 and 2
HHI	Hedgehog pathway inhibitor
HPV	Human papillomavirus
JBI	Joanna Briggs Institute
KC	Keratinocyte carcinoma
LOX	Lysyl oxidase
LVI	Lymphovascular invasion
MMP	Matrix metalloproteinase
MMS	Mohs micrographic surgery
NCCN	National Comprehensive Cancer Network
NMSC	Non-melanoma skin cancer
NOTCH	Notch signalling pathway
OS	Overall survival
PD-1/PD-L1	Programmed cell death protein 1/its ligand
PECO	Population–Exposure–Comparator–Outcome
PNI	Perineural invasion
PRISMA	Preferred Reporting Items for Systematic Reviews and Meta-Analyses
PTCH1	Patched 1 receptor
RT	Radiotherapy
SMO	Smoothened receptor
TIMP	Tissue inhibitor of metalloproteinases
TMB	Tumour mutational burden
TME	Tumour microenvironment
TP53	Tumour protein p53
UV	Ultraviolet radiation
