# Supplementary material for: Giant Keratinocyte Carcinomas: Clinical, Histopathological, Molecular and Health-System Factors with Therapeutic Implications—A Structured Narrative Review
Source: Cancers (Basel). 2026 Oct 18;18(20):3367. doi: 10.3390/cancers18203367 (PMC13702539; doi:10.3390/cancers18203367)
Supplement: Supplementary file 1 [file cancers-18-03367-s001.zip › cancers-4528653-supplementary.pdf]

Supplementary Materials: Giant Keratinocyte Carcinomas: Clinical, Histopathological, Molecular and Health-System Factors, with Therapeutic Implications—A Structured Narrative Review

Supplementary File S1 Retrospective methodological account

This account describes the revised structured narrative review with narrative synthesis. It is not a prospectively registered or independently time-stamped protocol. A record-level search and screening log is unavailable; historical selection counts cannot be verified and no PRISMA selection flow is reconstructed. During the present revision, the six SANRA domains—importance, aims, description of literature searching, referencing, scientific reasoning, and presentation of relevant data—were used as a retrospective reporting-quality self-check. This use of SANRA is not presented as a prospectively prespecified protocol or as a formal risk-of-bias tool.

Scope: clinical, histopathological, molecular, therapeutic and delayed-presentation evidence relevant to giant BCC and cSCC. Maximum diameter ≥5 cm is an operational harmonisation rule. Original thresholds and mixed-age secondary evidence are identified rather than converted into uniform adult-only cohorts.

Two principal evidence groups are used: direct giant-specific evidence and contextual evidence. Contextual sources include studies of advanced/high-risk disease and broader guideline, mechanistic, epidemiological or health-system literature. Directness does not imply high methodological quality.

Earlier drafts described a PubMed/MEDLINE/PMC publication window of 1 January 2015–31 March 2026. PMC overlaps PubMed/MEDLINE and is not treated as an independent source of unique records. Searches in other databases were mentioned but cannot be reconstructed from archived strings or logs. The earlier revision reported a focused update through 4 September 2026, adding eight giant-specific reports [11–18], and a reviewer-suggested contextual report [27]. These dates describe the prior revision record, not a newly executed search.

The final inventory contains 51 sources: nine direct [10–18] and 42 contextual. The methods-only PRISMA reference [31] is not counted. Each report appears once in both tables. Primary data and secondary case syntheses are not pooled; mixed sources are classified according to their direct clinical component while their general discussion remains contextual.

Source summaries are condensed, not a complete duplicate-extraction dataset. N/A means not applicable to that source type. Descriptions such as "institutional cohort" or "multiple studies" are retained source-summary labels, not numerical sample sizes. Missing numerical detail in these tables must not be interpreted as absence of reporting in the original publication. No individual JBI, AMSTAR-2 or AGREE-II scores are supplied or inferred.

Reconstructed PubMed search concepts

The following expression has balanced parentheses. It explains the concepts in the earlier manuscript; it is not an archived historical query, proof of execution, or a claim that it reproduces a previous result count.

(("basal cell carcinoma"[Title/Abstract] OR BCC[Title/Abstract] OR "cutaneous squamous cell carcinoma"[Title/Abstract] OR cSCC[Title/Abstract]) AND (giant[Title/Abstract] OR "locally advanced"[Title/Abstract] OR advanced[Title/Abstract]

OR unresectable[Title/Abstract] OR "high risk"[Title/Abstract])) OR (("basal cell carcinoma"[Title/Abstract] OR "cutaneous squamous cell carcinoma"[Title/Abstract]) AND ("Mohs surgery"[Title/Abstract] OR radiotherapy[Title/Abstract] OR vismodegib[Title/Abstract] OR sonidegib[Title/Abstract] OR cemiplimab[Title/Abstract] OR pembrolizumab[Title/Abstract] OR "perineural invasion"[Title/Abstract] OR "diagnostic delay"[Title/Abstract] OR "treatment delay"[Title/Abstract])) AND ("2015/01/01"[Date - Publication] : "2026/03/31"[Date - Publication])

Focused update concepts

("giant basal cell carcinoma"[Title/Abstract] OR "giant cutaneous squamous cell carcinoma"[Title/Abstract] OR ("giant squamous cell carcinoma"[Title/Abstract] AND (skin[Title/Abstract] OR cutaneous[Title/Abstract])))

The previous revision reports a literature check through 4 September 2026. The exact execution log and applied search filters are unavailable. This expression is therefore a reconstruction of reported concepts, not a new or independently verified update.

Source inclusion and interpretive limits

The retained 2013 review [32] is a contextual exception to the stated publication window. The giant-cSCC synthesis [17] is directly relevant to tumour size but includes mixed ages and is not an exclusively adult cohort. A source can include both primary clinical data and secondary discussion: Mott [10] is classified as direct for the case component. English- and Polish-language full texts were considered in the earlier search description; this restriction may have introduced language bias. Historical numbers screened or excluded and reason-specific exclusion counts are not reported because a case-by-case screening record is unavailable

https://doi.org/10.3390/xxxxx

Table S1. Concise characteristics and evidentiary roles.

Ref.	Source	Design	Disease / domain	Size definition	Sample / giant lesions	Selected treatment / outcome information	Directness
1	Peris et al., 2023	Guideline	BCC	Not size-specific	N/A	Management guideline	Contextual
2	Stratigos et al., 2023 Part 1	Guideline	cSCC	Not size-specific	N/A	Diagnostics/risk stratification	Contextual
3	Stratigos et al., 2023 Part 2	Guideline	cSCC	Not size-specific	N/A	Treatment guidance	Contextual
4	Schmults et al., 2023	Guideline	BCC	Not size-specific	N/A	NCCN management	Contextual
5	Schmults et al., 2021	Guideline	cSCC	Not size-specific	N/A	NCCN risk/treatment	Contextual
6	GBD 2021 Collaborators, 2024	Global modelling	NMSC	Not size-specific	Population	Burden estimates	Contextual
7	Pan et al., 2025	Global modelling	NMSC	Not size-specific	Population	Older-adult burden	Contextual
8	Zhou et al., 2025	Global modelling	Melanoma/NMSC	Not size-specific	Population	Burden trends	Contextual
9	Lin et al., 2025	Global modelling	Cancer/NMSC	Not size-specific	Population	General cancer burden	Contextual
10	Mott et al., 2017	Case report with narrative management review	Giant BCC	~≥5 cm	Case-based primary clinical evidence	Management features	Direct case; contextual discussion
11	Maimaiti et al., 2016	Retrospective surgical series	Giant BCC head/neck	≥5 cm	17 GBCC	Excision + reconstruction; 5–11 cm; surgical outcomes	Direct
12	Yazdani Abyaneh et al., 2017	Case-control + literature meta-analysis	Giant BCC	≥5 cm	13 GBCC (11 patients) + 26 controls	Infiltrative/morpheic phenotype, PNI, ulceration, growth; DSD predictors	Direct
13	Purnell et al., 2018	Comparative histopathology study	Giant vs conventional BCC	>5 cm	29 GBCC + 28 controls	Greater thickness, ulceration, infiltrative growth; histology not sufficient to explain size	Direct

Ref.	Source	Design	Disease / domain	Size definition	Sample / giant lesions	Selected treatment / outcome information	Directness
14	Vaca-Aguilera et al., 2019	Retrospective comparative cohort	Giant BCC	≥5 cm	115 GBCC among 5958 BCC	Mean 6.6 cm; longer duration; high-grade variants; head/neck 63%	Direct
15	Gualdi et al., 2020	Multicentre case series with controls	Giant BCC	>5 cm	59 GBCC + 458 controls	Clinico-histological correlates and factors associated with giant presentation	Direct
16	Oudit et al., 2020	Retrospective tertiary-centre cohort	Giant BCC	>5 cm	43 GBCC	Median 6.3 cm; delay correlated with size; treatment-specific recurrence; 2 disease-related deaths	Direct
17	van Dam et al., 2021	Systematic literature synthesis + case	Extra-anogenital giant cSCC	Giant/very large, size-defined case literature; mixed-age synthesis	42 well-described cases	Median delay 12 months; 64% head/neck; surgery 81%; DOD 13% among reported follow-up; not adult-only estimates	Direct; secondary synthesis
18	Mateuszczyk et al., 2024	Retrospective institutional series	Extra-anogenital giant cSCC	6–18 cm lesions	7 giant cSCC	71% head; all T3; nodal metastasis 42.9%; mostly surgery; selected systemic therapy	Direct
19	Que et al., 2018	Clinical review	Advanced cSCC	Not giant-specific	N/A	Advanced/high-stage management	Contextual
20	Monroe & Kakarala, 2021	Clinical review	Advanced head/neck BCC	Not giant-specific	N/A	Advanced BCC management	Contextual
21	Elias et al., 2021	Retrospective analysis	Melanoma	Different tumour	N/A	Delay factors only	Contextual
22	Lallas et al., 2021	Registry	Skin cancer/KC	Not giant-specific	Institutional	Pandemic diagnostic delay	Contextual
23	Bourkas et al., 2023	Systematic review/meta-analysis	Teledermatology	Not giant-specific	Multiple studies	Diagnostic agreement	Contextual
24	Mokos & Bašić-Jukić, 2021	Brief report	NMSC/transplant	Not giant-specific	Limited	Pandemic delay	Contextual

https://doi.org/10.3390/xxxxx

Ref.	Source	Design	Disease / domain	Size definition	Sample / giant lesions	Selected treatment / outcome information	Directness
25	Díaz-Calvillo et al., 2024	Systematic review/meta-analysis	Melanoma/KC	Not giant-specific	Multiple studies	Pandemic impact	Contextual
26	Seretis et al., 2021	Case-control	Skin cancer	Not giant-specific	Single setting	Treatment delay	Contextual
27	Gentileschi et al., 2020	Health-system/surgical pathway report	Skin cancer	Not giant-specific	Pandemic setting	Surgical reorganisation	Contextual
28	Combes et al., 2016	Clinical review	Facial BCC	Not giant-specific	N/A	Diagnostic/surgical context	Contextual
29	Słowińska et al., 2025	Retrospective study	Melanoma/NMSC	Not giant-specific	Institutional	Comorbidity context	Contextual
30	Khong et al., 2020	Clinical review	NMSC/RT	Not giant-specific	N/A	Radiotherapy context	Contextual
32	Lansbury et al., 2013	Systematic review/pooled observational	Non-metastatic cSCC	Not giant-specific	Multiple studies	Foundational treatment	Contextual
33	Motaparthi et al., 2017	Review	cSCC	Not giant-specific	N/A	AJCC8/pathology	Contextual
34	Crüts et al., 2025	Scoping review	cSCC/PNI	Not giant-specific	Multiple studies	PNI risk	Contextual
35	Ruiz et al., 2019	Retrospective cohort	cSCC	Not giant-specific	Institutional cohort	AJCC8 vs BWH	Contextual
36	Wysong, 2023	Clinical review	cSCC	Not giant-specific	N/A	General cSCC	Contextual
37	Stevens et al., 2023	Validation cohort	cSCC	Not giant-specific	Validation cohort	NCCN risk	Contextual
38	Trager et al., 2026	Review/update	cSCC	Not giant-specific	N/A	Current management	Contextual
39	Habashy et al., 2021	Narrative review	Advanced BCC/HHI	Not giant-specific	N/A	HHI mechanism/resistance	Contextual
40	Villani et al., 2022	Expert review	BCC/HHI	Not giant-specific	N/A	HHI resistance	Contextual

https://doi.org/10.3390/xxxxx

Ref.	Source	Design	Disease / domain	Size definition	Sample / giant lesions	Selected treatment / outcome information	Directness
41	Untaaveesup et al., 2025	Systematic review/meta-analysis	Advanced BCC	Not giant-specific	Multiple studies	HHI efficacy/toxicity	Contextual
42	Migden et al., 2018	Clinical trial	Advanced cSCC	Not giant-specific	Prospective	Cemiplimab	Contextual
43	Migden et al., 2020	Phase 2 trial	Locally advanced cSCC	Not giant-specific	Prospective	Cemiplimab	Contextual
44	Hoellwerth et al., 2025	Review	Advanced BCC	Not giant-specific	N/A	Advanced BCC therapy	Contextual
45	Jiang et al., 2024	Review	cSCC	Not giant-specific	N/A	Updated cSCC review	Contextual
46	Verkerk et al., 2024	Prospective real-world cohort	Advanced cSCC	Not giant-specific	Access-program cohort	Cemiplimab effectiveness	Contextual
47	Hughes et al., 2021	Phase 2 trial	Advanced cSCC	Not giant-specific	KEYNOTE-629	Pembrolizumab	Contextual
48	Stratigos et al., 2021	Phase 2 trial	Locally advanced BCC post-HHI	Not giant-specific	Prospective	Cemiplimab	Contextual
49	Chiang et al., 2023	Review	BCC/cSCC TME	Not giant-specific	N/A	Tumour microenvironment	Contextual
50	Gross et al., 2022	Phase 2 neoadjuvant trial	Stage II–IV cSCC	Not giant-specific	Prospective	Neoadjuvant cemiplimab	Contextual
51	Buruiană et al., 2024	Mechanistic review	SCC stroma	Not giant-specific	N/A	ECM/stroma	Contextual
52	van Lee et al., 2019	Retrospective cohort	Head/neck cSCC	Not giant-specific	Cohort	Mohs vs excision recurrence	Contextual

https://doi.org/10.3390/xxxxx

Table S2. Descriptive methodological limitations and directness. These are descriptive design-related considerations, not completed JBI, AMSTAR-2 or AGREE-II assessments. No tool-derived scores, formal certainty grades or quality weights are reported. Directness describes relevance to size-defined giant tumours; it is independent of study quality.

Ref.	Source	Appraisal considerations	Principal limitations	Directness	Use in narrative synthesis
1	Peris et al., 2023	Guideline-development domains; descriptive only	Guideline; not size-specific	Contextual	Inform guideline-based management; not giant-specific outcome evidence
2	Stratigos et al., 2023 Part 1	Guideline-development domains; descriptive only	Guideline; not size-specific	Contextual	Inform guideline-based management; not giant-specific outcome evidence
3	Stratigos et al., 2023 Part 2	Guideline-development domains; descriptive only	Guideline; not size-specific	Contextual	Inform guideline-based management; not giant-specific outcome evidence
4	Schmults et al., 2023	Guideline-development domains; descriptive only	Guideline; not size-specific	Contextual	Inform guideline-based management; not giant-specific outcome evidence
5	Schmults et al., 2021	Guideline-development domains; descriptive only	Guideline; not size-specific	Contextual	Inform guideline-based management; not giant-specific outcome evidence
6	GBD 2021 Collaborators, 2024	Observational/modelled-study domains	Ecological/modelled estimates; no giant-size stratification	Contextual	Provide context in the stated domain; no giant-specific effect estimate
7	Pan et al., 2025	Observational/modelled-study domains	Ecological/modelled estimates; no giant-size stratification	Contextual	Provide context in the stated domain; no giant-specific effect estimate
8	Zhou et al., 2025	Observational/modelled-study domains	Ecological/modelled estimates; no giant-size stratification	Contextual	Provide context in the stated domain; no giant-specific effect estimate
9	Lin et al., 2025	Observational/modelled-study domains	Ecological/modelled estimates; no giant-size stratification	Contextual	Provide context in the stated domain; no giant-specific effect estimate
10	Mott et al., 2017	Case-report and narrative-review considerations	Single-case clinical evidence; no comparative effect estimate; general treatment discussion is secondary	Direct case; contextual discussion	Illustrate giant-BCC presentation and management; no comparative inference

https://doi.org/10.3390/xxxxx

Ref.	Source	Appraisal considerations	Principal limitations	Directness	Use in narrative synthesis
11	Maimaiti et al., 2016	Observational-design domains; descriptive only	Observational; referral/selection bias; heterogeneous follow-up/definitions	Direct	Describe giant-specific observations; no pooled effect or certainty grade
12	Yazdani Abyaneh et al., 2017	Observational-design domains; descriptive only	Observational; referral/selection bias; heterogeneous follow-up/definitions	Direct	Describe giant-specific observations; no pooled effect or certainty grade
13	Purnell et al., 2018	Observational-design domains; descriptive only	Observational; referral/selection bias; heterogeneous follow-up/definitions	Direct	Describe giant-specific observations; no pooled effect or certainty grade
14	Vaca-Aguilera et al., 2019	Observational-design domains; descriptive only	Observational; referral/selection bias; heterogeneous follow-up/definitions	Direct	Describe giant-specific observations; no pooled effect or certainty grade
15	Gualdi et al., 2020	Observational-design domains; descriptive only	Observational; referral/selection bias; heterogeneous follow-up/definitions	Direct	Describe giant-specific observations; no pooled effect or certainty grade
16	Oudit et al., 2020	Observational-design domains; descriptive only	Observational; referral/selection bias; heterogeneous follow-up/definitions	Direct	Describe giant-specific observations; no pooled effect or certainty grade
17	van Dam et al., 2021	Review-design domains; descriptive only	Secondary case synthesis; mixed ages; incomplete outcome reporting and potential overlap with primary reports	Direct; mixed-age secondary synthesis	Describe giant-specific observations; no pooled effect or certainty grade
18	Mateuszczyk et al., 2024	Observational-design domains; descriptive only	Observational; referral/selection bias; heterogeneous follow-up/definitions	Direct	Describe giant-specific observations; no pooled effect or certainty grade
19	Que et al., 2018	Narrative/expert source appraisal	Secondary or expert source; no giant-specific effect estimate	Contextual	Provide context in the stated domain; no giant-specific effect estimate
20	Monroe & Kakarala, 2021	Narrative/expert source appraisal	Secondary or expert source; no giant-specific effect estimate	Contextual	Provide context in the stated domain; no giant-specific effect estimate

Ref.	Source	Appraisal considerations	Principal limitations	Directness	Use in narrative synthesis
21	Elias et al., 2021	Observational-design domains; descriptive only	Non-randomised; potential selection/confounding; generally not size-specific	Contextual	Provide context in the stated domain; no giant-specific effect estimate
22	Lallas et al., 2021	Observational-design domains; descriptive only	Non-randomised; potential selection/confounding; generally not size-specific	Contextual	Provide context in the stated domain; no giant-specific effect estimate
23	Bourkas et al., 2023	Review-design domains; descriptive only	Secondary synthesis; heterogeneity and/or indirectness	Contextual	Provide context in the stated domain; no giant-specific effect estimate
24	Mokos & Bašić-Jukić, 2021	Narrative/expert source appraisal	Secondary or expert source; no giant-specific effect estimate	Contextual	Provide context in the stated domain; no giant-specific effect estimate
25	Díaz-Calvillo et al., 2024	Review-design domains; descriptive only	Secondary synthesis; heterogeneity and/or indirectness	Contextual	Provide context in the stated domain; no giant-specific effect estimate
26	Seretis et al., 2021	Observational-design domains; descriptive only	Non-randomised; potential selection/confounding; generally not size-specific	Contextual	Provide context in the stated domain; no giant-specific effect estimate
27	Gentileschi et al., 2020	Narrative/expert source appraisal	Secondary or expert source; no giant-specific effect estimate	Contextual	Provide context in the stated domain; no giant-specific effect estimate
28	Combes et al., 2016	Narrative/expert source appraisal	Secondary or expert source; no giant-specific effect estimate	Contextual	Provide context in the stated domain; no giant-specific effect estimate
29	Słowińska et al., 2025	Observational-design domains; descriptive only	Non-randomised; potential selection/confounding; generally not size-specific	Contextual	Provide context in the stated domain; no giant-specific effect estimate
30	Khong et al., 2020	Narrative/expert source appraisal	Secondary or expert source; no giant-specific effect estimate	Contextual	Provide context in the stated domain; no giant-specific effect estimate
32	Lansbury et al., 2013	Review-design domains; descriptive only	Secondary synthesis; heterogeneity and/or indirectness	Contextual	Provide context in the stated domain; no giant-specific effect estimate
33	Motaparthi et al., 2017	Narrative/expert source appraisal	Secondary or expert source; no giant-specific effect estimate	Contextual	Provide context in the stated domain; no giant-specific effect estimate

https://doi.org/10.3390/xxxxx

Ref.	Source	Appraisal considerations	Principal limitations	Directness	Use in narrative synthesis
34	Crüts et al., 2025	Narrative/expert source appraisal	Secondary or expert source; no giant-specific effect estimate	Contextual	Provide context in the stated domain; no giant-specific effect estimate
35	Ruiz et al., 2019	Observational-design domains; descriptive only	Non-randomised; potential selection/confounding; generally not size-specific	Contextual	Provide context in the stated domain; no giant-specific effect estimate
36	Wysong, 2023	Narrative/expert source appraisal	Secondary or expert source; no giant-specific effect estimate	Contextual	Provide context in the stated domain; no giant-specific effect estimate
37	Stevens et al., 2023	Observational-design domains; descriptive only	Non-randomised; potential selection/confounding; generally not size-specific	Contextual	Provide context in the stated domain; no giant-specific effect estimate
38	Trager et al., 2026	Narrative/expert source appraisal	Secondary or expert source; no giant-specific effect estimate	Contextual	Provide context in the stated domain; no giant-specific effect estimate
39	Habashy et al., 2021	Narrative/expert source appraisal	Secondary or expert source; no giant-specific effect estimate	Contextual	Provide context in the stated domain; no giant-specific effect estimate
40	Villani et al., 2022	Narrative/expert source appraisal	Secondary or expert source; no giant-specific effect estimate	Contextual	Provide context in the stated domain; no giant-specific effect estimate
41	Untaaveesup et al., 2025	Review-design domains; descriptive only	Secondary synthesis; heterogeneity and/or indirectness	Contextual	Provide context in the stated domain; no giant-specific effect estimate
42	Migden et al., 2018	Clinical-trial appraisal	Mostly single-arm/non-randomised; no giant-size subgroup	Contextual	Inform advanced-disease therapy; no giant-specific treatment effect inferred
43	Migden et al., 2020	Clinical-trial appraisal	Mostly single-arm/non-randomised; no giant-size subgroup	Contextual	Inform advanced-disease therapy; no giant-specific treatment effect inferred
44	Hoellwerth et al., 2025	Narrative/expert source appraisal	Secondary or expert source; no giant-specific effect estimate	Contextual	Provide context in the stated domain; no giant-specific effect estimate
45	Jiang et al., 2024	Narrative/expert source appraisal	Secondary or expert source; no giant-specific effect estimate	Contextual	Provide context in the stated domain; no giant-specific effect estimate
46	Verkerk et al., 2024	Observational-design domains; descriptive only	Non-randomised; potential selection/confounding; generally not size-specific	Contextual	Provide context in the stated domain; no giant-specific effect estimate

https://doi.org/10.3390/xxxxx

Ref.	Source	Appraisal considerations	Principal limitations	Directness	Use in narrative synthesis
47	Hughes et al., 2021	Clinical-trial appraisal	Mostly single-arm/non-randomised; no giant-size subgroup	Contextual	Inform advanced-disease therapy; no giant-specific treatment effect inferred
48	Stratigos et al., 2021	Clinical-trial appraisal	Mostly single-arm/non-randomised; no giant-size subgroup	Contextual	Inform advanced-disease therapy; no giant-specific treatment effect inferred
49	Chiang et al., 2023	Narrative/expert source appraisal	Secondary or expert source; no giant-specific effect estimate	Contextual	Provide context in the stated domain; no giant-specific effect estimate
50	Gross et al., 2022	Clinical-trial appraisal	Mostly single-arm/non-randomised; no giant-size subgroup	Contextual	Inform advanced-disease therapy; no giant-specific treatment effect inferred
51	Buruiană et al., 2024	Narrative/expert source appraisal	Secondary or expert source; no giant-specific effect estimate	Contextual	Provide context in the stated domain; no giant-specific effect estimate
52	van Lee et al., 2019	Observational-design domains; descriptive only	Non-randomised; potential selection/confounding; generally not size-specific	Contextual	Provide context in the stated domain; no giant-specific effect estimate

Classification note: The classification of Mott [10] follows the primary case described in the publication. The adult-population limitation for van Dam [17] follows its mixed-age source population. These clarifications change interpretation and classification, not reported patient outcomes.
